# The Evaluation of an Integrated Tobacco Treatment Specialist in Primary Care

**DOI:** 10.1155/2023/9200402

**Published:** 2023-09-19

**Authors:** Rachel E. Miller, Jennifer M. Hill, Amanda F. Meyer

**Affiliations:** Mayo Clinic, 200 First Street SW, Rochester, MN 55905, USA

## Abstract

**Background:**

Primary care providers play a key role in screening for tobacco use and assessing desire to quit. Tobacco treatment specialists (TTS) are certified in helping patients who desire tobacco cessation. A primary care nurse practitioner within one Midwestern healthcare organization obtained TTS certification and integrated specialized tobacco cessation visits within a primary care clinic from February 2021 to February 2022.

**Purpose:**

To determine the efficiency and effectiveness of an integrated TTS-certified nurse practitioner (TTS-NP) in a primary care setting 1-year postimplementation.

**Method:**

This program evaluation utilized retrospective electronic health record review and included thirty-three patients. The logic model served as a framework to define efficiency and effectiveness.

**Results:**

Patients were referred by a provider (57.6%), nurse (15.2%), or self (27.3). Patients opted for in-person initial visits (81.8%) more than virtual (18.2%). Of a total of 73 scheduled visits, 8 (11%) were no-showed. Patients who self-referred had the lowest no-show rate (5.6%) compared to those referred by a provider (12.8%) or nurse (12.5%). Of the patients included, 87.9% set a goal quit date. Average time until first and second follow-up was 34.6 and 130.4 days after goal quit date. Follow-up was defined as the date of the patient's first message reply to the TTS-NP, or first visit following the goal quit date. A total of 51.9% (*n* = 14) and 63% (*n* = 17) reported cessation at the first and second follow-up. TTS-NP visit's cost, independent of any other coverage, was less than other specialty visits in primary care.

**Conclusion:**

TTS-NP visits in primary care enabled patients to benefit from lower cost and longitudinal follow-up within a familiar setting. Over half of patients achieved cessation. Results of this program evaluation suggest support for TTS-certified providers in primary care.

## 1. Introduction

Smoking tobacco is a significant problem that harms nearly every organ of the human body [1]. According to the Centers for Disease Control and Prevention, 34.2 million Americans reported currently smoking cigarettes in 2018, more than 16 million were living with a disease caused by smoking tobacco, and cigarette smoking was the leading cause of preventable death, causing more than 480,000 deaths per year [2]. Smoking tobacco resulted annually in more than $225 billion in direct medical care costs, yet only 2.7% of states' funds from tobacco taxes and court settlements were spent on smoking prevention or cessation programs [2]. While 68% of adult smokers expressed a desire to quit smoking, only 57.2% who had seen a health provider in the previous year reported receiving advice to quit [3].

Supporting patients and providing advice to quit is of utmost importance, as those who can achieve 1 week without smoking are 9 times more likely to successfully quit [4]. Therefore, it is essential that primary care providers are prepared to counsel people who smoke tobacco, such as through utilization of the “5 A's” method [5]. Healthcare providers must explore strategies to assist patients in considering a quit attempt, creating a plan to quit, and achieving their goals for smoking tobacco cessation. There remains room for improvement in providing tobacco treatment for adults seeking assistance with smoking cessation.

To close this gap in tobacco cessation treatment, it is imperative to implement evidence-based interventions. One such way for healthcare providers to build competence is to receive tobacco treatment specialist (TTS) certification. Programs for TTS certification provide nationally standardized, evidence-based competencies, knowledge, and skills for providers supporting patients who are attempting tobacco cessation [6]. These programs further providers' understanding of the determinants of tobacco use disorder, including biological, psychological, and social aspects; examine the physical and behavioral health impact of tobacco use; explore best practices for supporting and treating patients with tobacco use disorder; and discuss pharmacotherapy, counseling skills, and cognitive and behavioral strategies to assist tobacco users in quitting [7]. Certification as a TTS signifies competency in tobacco dependence knowledge and education, counseling skills, assessment interviews, treatment planning, documentation, pharmacotherapy, relapse prevention, and specific law and ethics regarding treatment for tobacco dependency [8]. A TTS is particularly equipped to overcome barriers of tobacco cessation counseling in the primary care setting such as provider competence, personal attitudes, and comfort level with tobacco treatment [9]. In primary care, it is essential that providers are prepared to assess and provide tobacco cessation counseling adequately and efficiently.

## 2. Methods

At one primary care clinic in a Midwestern health system, a primary care nurse practitioner (NP) sought to improve patients' tobacco cessation treatment and thus obtained TTS certification. Once certified, the NP initiated education within the multidisciplinary team regarding the ability to integrate tobacco cessation consults in the primary care clinic. Prior to integration of the TTS-certified NP (TTS-NP) into the primary care clinic, all patients were required to visit a separate facility for nicotine dependence within the organization to receive specialized tobacco treatment care with a TTS. Visits incorporating the TTS-NP in primary care began in February 2021 and continued through February 2022.

Previous research of this program studied the perceptions of stakeholders at the participating clinic, including physicians, physician assistants, NPs, registered nurses, and licensed practical nurses [9]. A cross-sectional survey investigated stakeholders' insights regarding familiarity with the TTS-NP role, utilization of TTS-NP referral, barriers to utilization, value of the TTS-NP visit interaction, and benefits of an on-site TTS-NP. Results reinforced the positive impact of a TTS-NP in the primary care setting as perceived by the multidisciplinary care team, as 55% reported utilizing the TTS-NP for direct patient care and the majority strongly agreed that utilizing the TTS-NP was valuable [9]. The purpose of this project was to supplement previous research of the program and evaluate utilizing the following question: how efficient and effective was an integrated TTS-NP in a primary care setting 1-year postimplementation?

### 2.1. Design

This program evaluation was implemented to assess the efficiency and effectiveness of the process for TTS-NP visits integrated in one primary care clinic. By utilizing retrospective electronic health record (EHR) review, this program evaluation's primary outcome included measures of efficiency and effectiveness, with smoking cessation rates being a secondary measure included within the evaluation of effectiveness. The logic model served as a framework for defining effectiveness and efficiency. The logic model is a planning tool which illustrates how a program is expected to produce results by presenting the resources available, activities carried out, expected outcomes or results of the project, and impact of the program [10]. Process variables define efficiency, and outcome variables define effectiveness. The logic model also considers “moderators,” which are contextual factors that are beyond the program's control but might help or hinder achievement of program outcomes [10].

### 2.2. Setting and Sample

The setting for the TTS-NP visits was one of five ambulatory primary care clinics within a large Midwestern tertiary healthcare center. The involved primary care clinic is located on the outer edge of a city with a population of over 100,000 people. This clinic utilizes a multidisciplinary team-based approach to care for patients by integrating providers and staff from pediatrics, internal medicine, family medicine, pharmacy, and subspecialties. It employs the patient-centered medical home model, which allows increased communication between behavioral health specialists and primary care team members by having the entire team colocated. The advantage of this clinic is that patients do not have to drive into the heart of the city to receive care at the main multicomplex healthcare center.

Data from patients 18 years of age or older who completed an initial visit for smoking tobacco cessation with the TTS-NP after integration in primary care in February 2021 were included in the analysis. Patients were excluded from analysis if they visited with the TTS-NP for cessation of chewing tobacco. Patients were included in the analysis of smoking cessation rates if they met criteria and had EHR documentation of a set goal quit date and two documented follow-up timepoints. Follow-up timepoints included communication between the patient and any healthcare provider or nurse that discussed tobacco cessation status, either by means of an in-person visit, virtual (telephone or video) visit, or patient online service portal message. For patients who were enrolled in patient online services, the TTS-NP sent a portal message on the goal quit date with an open response to enable patients to send a message reply, and follow-up was defined as the date of the patient's first reply mentioning cessation status. For patients who did not utilize online services, follow-up was defined as the date of first in-person or virtual visit following the goal quit date. Cessation was defined as patients' self-reported abstinence from smoking any cigarettes between their goal quit date and the date of their follow-up portal message or visit. The Institutional Review Board at Viterbo University and Mayo Clinic approved this study.

### 2.3. Process Evaluated

For the TTS-NP visits, the process began when a patient was referred by another care provider or registered nurse, or when a patient self-referred by contacting the clinic with an interest in tobacco cessation. The patient was then added to the TTS-NP calendar, and the appointment scheduling team coordinated with the patient to schedule the TTS-NP visit. The visits were integrated into the normal schedule template for the TTS-NP. Patient visits were typically limited to a 30-minute appointment slot. TTS-NP visit times were shorter compared to 60-minute visits at the specialized clinic for nicotine dependence within the organization and 40- and 60-minute visits for other integrated collaborative specialties (such as cardiology and endocrinology) within the primary care clinic. During the appointment, the TTS-NP assessed the patient's tobacco dependence and utilized motivational interviewing, behavioral counseling, and shared decision-making techniques to assist the patient in developing a personalized plan for smoking cessation, which could include initiation of prescription medication and/or the patient setting a goal quit date for smoking cessation.

### 2.4. Data Collection

All data to evaluate efficiency and effectiveness of the TTS-NP visits in primary care was collected retrospectively via EHR review. Demographic data was confirmed by the TTS-NP at the time of the patient visit and included patient age, years smoked, packs per day, motivation and confidence self-ratings on a 0-10 scale, number of previous quit attempts, presence of cooccurring mental health or substance use disorders, and longest previous quit attempt.

#### 2.4.1. Efficiency Metrics

Within the logic model framework, efficiency is measured by process variables, including inputs, activities, and outputs. “Inputs” are resources necessary for implementation (organizational support for integration of TTS-NP, financial cost of TTS-NP certification, and TTS-NP visits); “activities” are the actions performed by the program personnel using those resources (care team education, referral of patients, and scheduling and conducting TTS-NP visits); and “outputs” are products or deliverables that result from the activities (documentation of visit, follow-up visits, and no-shows) [10]. Therefore, evaluation of efficiency involved collection of data such as visit cost, including how it compared to primary care specialty visits and to visits at the specialized nicotine dependence clinic within the same organization; cost for the TTS-NP to complete the TTS certification; referral process, including source of referral and time elapsed between date of referral and initial TTS appointment; average amount of time that passed between initial appointment, first follow-up, and second follow-up; format of visits (in-person or virtual); no-show rates, including how those varied according to referral source; and percentage of patients lost to follow-up after initial visit.

#### 2.4.2. Effectiveness Metrics

Within the logic model, effectiveness is defined by program “outcomes,” including the short- and intermediate-term changes that occur in people or conditions because of activities and outputs [10]. Evaluation of long-term changes was outside the scope of this program evaluation. Measures of effectiveness included the total number of patients who were referred to the TTS-NP and who met program evaluation inclusion criteria; total number of appointments scheduled with the TTS-NP while the program was active; steadiness of visit demand, defined by the total number of new patient visits completed across three 18-week intervals of time; percentage of patients who set goal quit dates; relationship between patients' confidence and motivation self-ratings at first and second follow-up; smoking cessation rates at the first two follow-up timepoints; relationship between cessation at first and second follow-up; and total number of weeks the TTS-NP program was active. Only those who set a goal quit date during their initial TTS visit and had two follow-up timepoints documented mentioning smoking status were included in the smoking cessation outcomes.

### 2.5. Data Analysis

Evaluation of demographic, efficiency, and effectiveness data included descriptive statistics. All statistics were calculated utilizing Microsoft Excel data analysis and Analysis ToolPak. The Excel “count” feature was utilized to tally the total number of patients who visited with the TTS-NP and met study inclusion criteria, the total number of appointments scheduled with the TTS-NP, and the number of visits in each 18-week period. Percentages were calculated in Excel to analyze the proportion of patients who set goal quit dates, no-show rates, visit formats, longest previous quit attempt, referral sources, and smoking cessation rates at the first and second follow-up timepoints. Excel data analysis was utilized to calculate the mean, standard error, *t*-distribution confidence level, and 95% confidence interval for average amount of time elapsed between referral and initial visit and average amount of time that passed between initial appointment, first follow-up, and second follow-up.

Data reflecting whether a patient did or did not achieve cessation was coded numerically to assess the degree of relationship between cessation at first and second follow-up. Cessation was coded into ordinal values, where a value of 1 indicated “yes” and 2 indicated “no,” and an *r*-value was calculated. The same method was utilized to measure correlation between patients' confidence and motivation self-ratings with cessation at first and second follow-up. Cost estimates were calculated by utilizing the healthcare organization's cost estimator tool. The Current Procedural Terminology (CPT) billing codes for primary care-based TTS-NP visits, primary care-based visits in other specialties, and TTS visits in the nicotine dependence specialty clinic were utilized to estimate cost of professional charges without any insurance coverage or uninsured discounts.

## 3. Results

A total of 36 patients were referred to the TTS, but a total of 33 patients met inclusion criteria, as three patients were excluded due to being referred for chewing tobacco cessation. Of the 33 patients who met inclusion criteria, 45.5% were male (*n* = 15) and 54.5% were female (*n* = 18). Other demographic characteristics analyzed included age, number of years smoked, packs per day smoked, number of previous quit attempts, and self-rated levels of motivation and confidence levels on a scale from 0 to 10 (see Table 1). Additional demographic data included longest previous quit attempt and presence of cooccurring mental health and/or substance use disorder (see Table 2).

### 3.1. Efficiency

Cost was one input measured in the evaluation of efficiency, as it reflects the financial resources necessary to implement the TTS-NP visits. Cost of the TTS certification program for the TTS-NP was $1,000 for the intensive, 5-day course and included all course materials, pre- and postcourse testing, certificate of competency upon completion, and access to the education program website [11]. Further cost evaluation included an analysis of cost comparisons between various types of visits.  Table 3 provides a comparison of the cost and duration of TTS-NP visits against other specialty visits. Comparison of the cost of TTS-NP visits to other specialty consults in primary care is useful because the TTS-NP provided a specialist visit but did not bill as a specialty service. Comparison to the nicotine dependence specialty clinic is useful given that this compares the cost of visits with a TTS across two settings within the same organization.

Evaluation of the TTS-NP visits revealed that a total of 57.6% (*n* = 19) patients were referred to visit with the TTS-NP by another provider (physician, NP, or PA), while 15.2% (*n* = 8) were referred by a nurse, and 27.3% (*n* = 9) self-referred. No-show rates varied based upon referral source, as depicted in Table 4. Most of the TTS-NP initial visits were completed in-person (81.8%, *n* = 27) compared to virtually (18.2%, *n* = 6). The mean time between referral and initial appointment with the TTS-NP was 13.8 days (95% CI [8.8, 18.7]). While the TTS-NP sent a message to patients on their goal quit date, many patients waited to respond or to request their follow-up visit. The average time between goal quit date and first follow-up from the patient was 34.6 days (95% CI [15.6, 53.7]), and average time between goal quit date and second follow-up was 130.4 days (95% CI [84.9, 175.8]). Out of 73 visits scheduled, a total of 65 visits were completed, equating to an 11% no-show rate. Six patients (18.2%) were lost to follow-up, defined as no documentation in the EHR of smoking cessation completed after their initial TTS-NP visit.

### 3.2. Effectiveness

A total of 33 patients participated in the TTS-NP visits in the primary care clinic for smoking cessation between February 2021 and February 2022, and a total of 65 visits were completed and billed by the TTS-NP; this does not account for patient online service portal messages from the TTS-NP, as these were not billed encounters. The TTS-NP visits within the primary care clinic were active for a total of 54 weeks, and when dividing into three 18-week intervals, there was a steady rate of patient visits. There were 10, 12, and 11 patients seen during the first, second, and third 18-week intervals, respectively.

Of all the participating patients, 87.9% (*n* = 29) set a goal quit date, and 81.8% (*n* = 27) were included in the smoking cessation rate analysis as they had at least two follow-up timepoints documented by a healthcare provider in the EHR after the goal quit date. The TTS-NP was the responsible provider for 70.4% (*n* = 19) and 48.1% (*n* = 13) of the first and second follow-up timepoints, respectively; the remainder were completed and documented by another healthcare provider, such as the patient's primary care provider, another specialty provider, or a nurse.

A total of 51.9% (*n* = 14) of patients achieved smoking cessation at the first follow-up, whereas 63% (*n* = 17) achieved smoking cessation at the second follow-up. An analysis of the correlation between variables evaluating effectiveness is shown in Table 5. There was a moderately strong statistically significant correlation between smoking cessation at the first and second follow-up timepoints, as well as between self-rated motivation and cessation at the second follow-up.

## 4. Discussion

This program evaluation sought to answer the following question: how efficient and effective was an integrated TTS-certified NP in a primary care setting 1-year postimplementation? Overall, the integration of TTS visits within primary care had efficient and effective outcomes, and findings support benefit of reinitiating TTS-NP visits within primary care.

### 4.1. Efficiency

The cost of patient visits with the TTS-NP was lower than other specialist visits within the primary care clinic and within the specialty clinic for nicotine dependence. This lower cost reduces financial burden for not only the patient but also the healthcare organization and the broader healthcare system. Because the TTS-NP could visit with patients, prescribe, and bill independently, this addressed common barriers in the primary care setting, such as physician time and reimbursement issues [9]. Consequently, this may support the notion that healthcare organizations should consider covering the cost of TTS certification for NPs and other healthcare providers in order to increase patient access and reduce cost for patients and the healthcare system.

Additionally, the TTS-NP visits demonstrated efficiency because the no-show rate (11%) was below the average clinic no-show rate of 18.8% [12]. A lower no-show rate positively impacts delivery and cost of care and resource planning. Additionally, an overwhelming majority (81.8%) of patients preferred to meet with the TTS-NP in-person rather than virtually, which strengthens the relationship made between provider and patient. While telehealth visits for tobacco cessation can still provide benefit for patients, in-person visits enabled the TTS-NP to physically assess the patient and eliminated the difficulties that may arise with virtual visits, such as technical difficulties or lack of access to internet or video visit software. Additionally, health insurance coverage of telemedicine appointments varies by state and insurance provider, which has the potential to impact efficiency of virtual visits.

There was also demonstrated efficiency among the multidisciplinary care team in utilizing the TTS-NP, given 75% (27/36) of the total referrals were from another physician, NP, PA, or nurse. Lastly, the TTS-NP visits demonstrated efficiency in patient follow-up, as 81.8% had at least two follow-up timepoints documented, showing that a majority of patients had longitudinal care within their primary care setting, where they reported feeling satisfied with the convenience of scheduling and comfort or familiarly with clinic staff and feeling confident with their primary care provider giving personal recommendations [9].

### 4.2. Effectiveness

The integration of TTS visits in primary care was effective with regard to maintaining a steady number of visits throughout the entirety of the 54 weeks during which it was active. The TTS-NP was effective in working with patients to set goal quit dates, as almost 90% of the patients succeeded in doing so. According to the United States Department of Health and Human Services (DHHS), an average of 47.1% of cigarette smokers had attempted to quit smoking between 2012 and 2014 [13]. Therefore, the rate of smoking cessation attempts among patients who visited with the TTS-NP was 1.8 times higher than those previously surveyed by the DHHS.

Duration of time elapsed between goal quit date and first and second quit date was impacted by the patients' promptness of message reply, as well as the limited number of TTS-NP visit slots. The smoking cessation rates among patients who visited with the TTS-NP in primary care were above the reported cessation rate of the specialty clinic, with 52% and 63% achieving smoking cessation at the first and second follow-up timepoints, respectively. The specialty clinic location advertises “the 6-month stop rate among outpatients who receive a tobacco treatment specialist consult … is over 25 percent” [14]. Over half of the patients visiting with the TTS-NP in primary care achieved cessation at their follow-ups, and cessation rates were greater than those advertised by the specialty clinic. A true comparison of cessation rates between patients who met with the TTS-NP and national averages is not achievable because there is no current, well-defined national average rate of smoking cessation. The data obtained as part of this evaluation may enable other providers to make direct comparisons to smoking cessation data from their own institutions and processes.

## 5. Limitations

A limitation is that data from retrospective EHR review was manually studied and entered by one researcher, so there is possibility for human error. For example, as patient online service portal messages were not billed or tallied on the TTS-NP calendar, it is possible that some follow-up may have been inadvertently missed. Another limitation is that this study only evaluated one TTS at one specific primary care setting. Personalities and clinic environment impact the ability to generalize this to other primary care sites. Lastly, staffing challenges and the SARS-CoV-2 pandemic were moderators to the longevity of the TTS-NP visits. Staffing challenges amid the pandemic led to a reduction in the available weekly TTS-NP visit slots, and subsequently, the TTS-NP transitioned to a new professional role.

## 6. Conclusion

This program evaluation revealed numerous benefits of integration of a TTS-NP in the primary care clinic and supported its efficiency and effectiveness. The cost of TTS-NP visits was lower than the cost of other specialty visits, which benefits both the patient and the healthcare system. Improved cessation rates revealed that patients were well-supported in their journeys toward smoking cessation when patients visited with the TTS-NP. Longitudinal follow-up with patients led to improvements in smoking cessation rates over time. Because primary care providers commonly follow their patients for a prolonged period, this enables improved follow-up for tobacco smoking cessation. While further research is needed to understand the patients' long-term cessation rates and perceptions of the TTS-NP visits, the results of this program evaluation provide support for a TTS-certified provider in the primary care clinic and may encourage more primary care providers to obtain specialty certification to improve access to care and patient outcomes.

## Figures and Tables

**Table 1:** Average sample population data.

	Mean (range)	95% CI	
		LL	UL
Age	43.9 (20-67)	39.0	48.9
Years smoked	24.8 (5-50)	20.3	29.3
Packs per day	0.8 (.25-2)	0.7	0.9
Number of previous quit attempts	2.7 (0-7)	1.8	3.6
Motivation self-rating	7.9 (0-10)	7	8.8
Confidence self-rating	5.8 (0-10)	4.7	6.9

**Table 2:** Prevalence of cooccurring disorders and longest previous quit attempt.

		Total (*N* = 33) *n* (%)
Cooccurring disorder(s)	Mental health	15 (45.5)
	Substance use	8 (24.2)

Longest previous quit attempt	None	1 (3.0)
	1 month or less	6 (18.2)
	2-6 months	9 (27.3)
	7-12 months	7 (21.2)
	More than 1 year	6 (18.2)
	No data available	4 (12.1)

**Table 3:** Cost comparisons.

Visit type	Duration	CPT code	Total estimated professional charges (without insurance)
TTS in primary care	30 min	99214	$290
Other specialty consults in primary care	40 min	99243	$457
	60 min	99244	$650
Nicotine dependence specialty clinic	60 min	99204 and 99407	$588
	1 week	99412	$6,389

**Table 4:** No-show rates by source of referral.

Referral source	*n*	Total visits scheduled	Total visits completed	Total visits no-showed	Percentage no-showed
Provider	19	47	41	6	12.8%
Nurse	5	8	7	1	12.5%
Self	9	18	17	1	5.6%
Total	33	73	65	8	11.0%

**Table 5:** Correlation between variables evaluating effectiveness.

Variable		*r*	*p*
1	2		
Cessation at first follow-up	Cessation at second follow-up	0.49	.01^∗^

Self-rated confidence	Cessation at first follow-up	0.04	.87
	Cessation at second follow-up	0.32	.16

Self-rated motivation	Cessation at first follow-up	0.12	.60
	Cessation at second follow-up	0.47	.02^∗^

^∗^ *p* < .05.

## Data Availability

All data has been provided in the results.
